# Relationship of health workers with their organization and work: a cross-cultural study

**DOI:** 10.1590/S1518-8787.2016050006285

**Published:** 2016-04-27

**Authors:** Montserrat Yepes-Baldó, Marina Romeo, Rita Berger

**Affiliations:** I Grupo de Investigación Consolidado en Psicología Social, Ambiental y Organizacional. Universidad de Barcelona. Barcelona, España; II Departamento de Psicología Social. Facultad de Psicología. Universidad de Barcelona. Barcelona, España

**Keywords:** Personnel Administration, Hospital, Patient Care Team, Working Conditions, Cultural Characteristics

## Abstract

We analyzed the differences, by Student’s t-test and ANOVA, between nurses and physicians from Portugal, Poland, Spain, and United Kingdom regarding their relationship with their work and organization. In total, 1,401 professionals answered the HSA-QHPR questionnaire. There are different levels of connection between physicians and nurses. The United Kingdom has the lowest levels of connection with the work while Portugal has the highest levels of relationship with the organization. The results provide guidelines for the development of policies and differential strategies aimed at improving the quality of healthcare service.

## RESUMEN

Se analizan las diferencias, mediante *t* de Student y ANOVA, entre personal de enfermería y médicos de Portugal, Polonia, España y Reino Unido en cuanto a su relación con el trabajo y con la organización. En total, 1.401 profesionales contestaron el cuestionario HSA-QHPR. Existen niveles diferentes de vínculo entre médicos y personal de enfermería. En Reino Unido se obtienen los niveles más bajos de vínculo con el trabajo, y en Portugal los niveles más altos de relación con la organización. Los resultados proporcionan guías para la elaboración de políticas y estrategias diferenciales orientadas a la mejora de la calidad del servicio asistencial.

## INTRODUCTION

The healthcare sector has experienced significant changes during the years of economic crisis. Although it has been one of the few sectors in which jobs have been created^a^, the working conditions of health workers have worsened: frozen wages, reduction in personnel, work overload, increased staff turnover, decreased status, and reduced work expectations^1^.

This context causes professionals to hold on to their current jobs, since they need to keep them^a^. However, to maintain the quality of care standards required in this sector, the conditions necessary to achieve a greater engagement of employees with their work and their organization should be created, thus avoiding the current high personnel turnover rates^b^.

The connection between employees and their work will be narrower if they feel they are willing to strive in their work (motivation), and their performance does not cause them strain or anxiety (stress), emotional exhaustion, cynicism, or lack of fulfillment at work (burnout)^c^.

On the other hand, the connection that employees establish with their organization will increase as they feel that they have influence on decision making (participation), develop a psychological connection with their organization (commitment), identify themselves as its members, are proud to belong to it and wish to remain members of it (identification), are satisfied, have a shared positive response regarding the organizational life (climate), and realize the existence of transformational leaders (leadership)^c^.

Faced with this situation, and based on the model and measuring instruments of the Human System Audit Quality of Human Processes and Resources (HSA-QHPR)^c^, our objective was to examine whether there are differences between nurses and physicians in four European countries regarding their relationship with their work and organization.

## METHODS

This study is a correlational design. It included a sample of 1,401 healthcare professionals in Portugal, Poland, Spain, and United Kingdom for three weeks. The average participation rate was 29.5% (sampling error = 2.38). A total of 902 participants were nurses (58.9%) and 156 were physicians (11.1%), while the remaining 30.0% were non-medical personnel.

The HSA-QHPR (Human System Audit - Quality of Human Processes and Resources) questionnaire included 87 items classified into six categories regarding the individual’s relationship with their work (72 items) and with their organization (15 items). In addition, they included variables related to the country and the professional activity.

The categories comprising the dimension that assesses the person-organization relationship were: “Participation” (5 items. Example: *I think that the level of participation in this hospital is effective),* “Leadership” (8 items. Example: *Our boss creates ways to motivate us),* “Identification” (6 items. Example: *I feel proud when I tell others that I work at this hospital),* “Commitment” (12 items. Example: *An important reason why I am still working at this hospital is that I feel other hospitals cannot offer me better compensations),* “Climate” (33 items. Example: *Here, the staff shares and positively welcomes the vision and objectives of the hospital)*, and “Satisfaction” (8 items. Example: *I’m satisfied with the employee benefits that this hospital offers – meal vouchers, pension plan, life insurance, etc.).*

The dimension that assesses the person-work relationship included: “Burnout” (6 items. Example: *I feel emotionally exhausted),* “Stress” (3 items. Example: *I feel tense about my work),* “Activation” (3 items. Example: *I feel full of energy at work),* “Motivation” (3 items. Example: *I’m willing to push myself at work).* All items are measured on a Likert scale (1 = strongly disagree; 5 = strongly agree).

To confirm the two-dimensional structure of the HSA-QHPR instrument and the internal consistency of the scales, several confirmatory factor analyses were conducted (adjusted goodness of fit index [AGFI] ≥ 0.9, normed fit index [NFI] ≥ 0.9, and standardized root mean square residual [SRMR] ≤ 0.15) , and Cronbach’s α (α ≥ 0.65). The differences between nurses and physicians were confirmed by Student’s t-test and between countries based on ANOVA (p ≤ 0.05).

The study had the approval of the research ethics committees of all participating centers. All participants received a letter explaining the procedure and objectives of the research, ensuring the confidentiality of their data and anonymity, as well as their right to leave the study at any time without negative consequences for them.

## RESULTS

The factor analysis confirmed the two-dimensional structure of the HSA-QHPR instrument (adjusted goodness of fit index (AGFI) = 0.904 - 0.965, normed fit index (NFI) = 0.978 - 0.992, and standardized root mean square residual (SRMR) = 0.029 - 0.125). Likewise, the internal consistency of the scales (α between 0.693 and 0.937) was confirmed.

The Figure shows that participants had higher levels of relationship with their work than with their organization (nurses: t = 12.07, p < 0.001; physicians: t = 8.52, p < 0.001). In addition, we observed significant differences between them regarding the organization, of which nurses obtained the highest levels. Also, there are significant differences between countries regarding the levels of relationship of employees with their work and their organization (person-work: F = 11.69, p < 0.001; person-organization: F = 42.14, p < 0.001). The United Kingdom had the lowest levels of relationship of employees with their work. However, regarding the levels of relationship of employees with the organization, it was below Portugal and above Spain and Poland.

**Figure:**
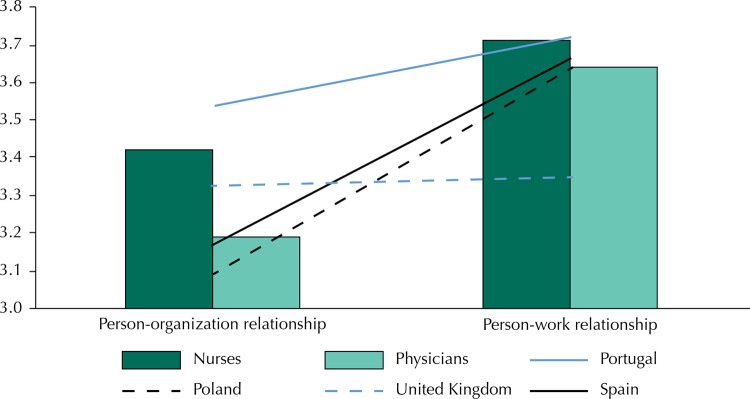
Comparisons of the levels of relationship with the work and the organization by countries and groups.

Portugal had the highest levels of relationship of employees with their organization compared to other countries. Finally, we did not find any significant differences between Spain and Poland.

## DISCUSSION

The current economic crisis, and the job insecurity resulted from it, creates the need for in-depth knowledge of the relationship of healthcare professionals with their work and their organizations. Thus, the results obtained in this research showed the existence of different levels of connection between physicians and nurses in four European countries. Both groups had a greater connection with their work than their organization, i.e., they view their relationship with the work more important than their relationship with the organization.

However, nurses showed a greater connection with their organization in all countries, except the United Kingdom. In addition, we did not find any significant differences between the connection established by physicians and nurses with their work and their organization in this country. The United Kingdom is traditionally one of the countries that receives more personnel from other countries of the European Union, and even from outside it^d^. This can lead to difficulties for immigrant professionals regarding the adaptation to the job and knowledge of the operation of the British healthcare system. The results obtained would be product of such a situation, since the professionals would resemble both dimensions, considering them as a single one.

On the other hand, Portugal had higher levels regarding work and organization, although these levels were statistically different only regarding the person-organization relationship when compared with the other countries.

The non-existence of statistically significant differences between Poland and Spain seems quite relevant, since the situation of the healthcare sector in both countries shows completely opposite trends. While in Spain, the crisis has led to a worsening of the working conditions of health workers, in Poland, we observed the return of migrants, due to the improvement of the working conditions compared to their countries of destination^b,d^.

As limitations of the study, we should highlight that samples correspond to hospitals from four European countries and that, therefore, results must be verified in other cultural contexts. In addition, we consider appropriate, in future studies, to use other instruments other than the questionnaire, and include efficiency criteria of the organization to avoid the risk of generating spurious correlations by the method of common variance^2^. Despite such limitations, the results obtained provide guidelines for the development of policies and strategies tailored to the needs of both occupational profiles in different countries, aiming at improving the quality of the healthcare service and decreasing personnel turnover.

## Footnotes

a European Commission. Toward a job-rich recovery: edición electrónica. Bruselas; European Comission; 2012 [cited 2015 Feb 19]. Available from: http://ec.europa.eu/social/main.jsp?catId=89&langId=en&newsId=1270&moreDocuments=yes&tableName=news

b European Commission. The EU single market: regulated professions database: edición electrónica. Bruselas; European Comission; 2011 [cited 2015 Feb 19]. Available from: http://ec.europa.eu/internal_market/qualifications/regprof/

c Yepes-Baldó M. El constructo psicosocial de calidad de los procesos y recursos humanos: desarrollo teórico y validación empírica [tesis]. Barcelona: Universitat de Barcelona; 2010 [cited 2015 Feb 19]. Available from: http://tdx.cat/bitstream/handle/10803/77653/MYB_TESIS.pdf?sequence=1

d Buchan J, Wismar N, Glinos IA, Bremmer J, editors. Health professional mobility in a changing Europe: new dynamics, mobile individuals and diverse responses. Copenhagen: World Health Organization; European Observatory on Health Systems and Policies; 2014 [cited 2015 Feb 19]. Available from: http://www.euro.who.int/_data/assets/pdf_file/0006/248343/Health-Professional-Mobility-in-a-Changing-Europe.pdf?ua=1
